# Obesity as a Possible Risk Factor for Lost-time Injury in Registered Nurses: A Literature Review

**DOI:** 10.1016/j.shaw.2014.12.006

**Published:** 2015-01-12

**Authors:** Gillian Jordan, Behnam Nowrouzi-Kia, Basem Gohar, Behdin Nowrouzi

**Affiliations:** 1 School of Occupational and Public Health and Safety, Ryerson University, Toronto, Ontario, Canada; 2 Michael G. Degroote School of Medicine, McMaster University, Hamilton, Ontario, Canada; 3 Centre for Research in Occupational Safety and Health, Laurentian University, Sudbury, Ontario, Canada

**Keywords:** registered nurses, time-loss injuries, work disability prevention, workplace, occupational injury

## Abstract

Time-loss injuries are still a major occurrence in Canada, injuring thousands of Canadian workers each year. With obesity rates on the rise across the country, as well as around the world, it is important that the possible effects of obesity in the workplace be fully understood, especially those effects linked to lost-time injuries. The aim of this paper was to evaluate predictors of workplace lost-time injuries and how they may be related to obesity or high body mass index by examining factors associated with lost-time injuries in the health care sector, a well-studied industry with the highest number of reported time loss injuries in Canada. A literature review focusing on lost-time injuries in Registered Nurses (RNs) was conducted using the keywords and terms: lost time injury, workers' compensation, occupational injury, workplace injury, injury, injuries, work, workplace, occupational, nurse, registered nurse, RN, health care, predictors, risk factors, risk, risks, cause, causes, obese, obesity, and body mass index. Data on predictors or factors associated with lost-time injuries in RNs were gathered and organized using Loisel's Work Disability Prevention Management Model and extrapolated upon using existing literature surrounding obesity in the Canadian workplace.

## 1 Introduction

Occupational illness and injury remain important issues in today's workplace. The International Labor Organization states that each year it is estimated that 313 million accidents resulting in worker injury occur in workplaces around the world, equivalent to 160 workers injured every 15 seconds [1]. In Canada, approximately one million occupational injury claims are reported to provincial and territorial governments each year across the country [2]. In 2012, > 245,000 of these applications were accepted as lost-time injuries by worker compensation (WC) boards across Canada for compensation [3]. These injuries directly cost the Canadian economy approximately 9.7 billion dollars in 2008, with more current estimates of annual costs expected to exceed 19 billion dollars in 2010 [2].

Of the 245,000 lost-time injuries compensated in Canada in 2012, > 41,000 were reported by the health care industry, more than any other identified industry in Canada [3]. The health care industry employs 10% of Canada's working population, accounting for > 1.6 million Canadian jobs in 2006 [4]. In 2013, the Canadian Federation of Nurses Unions reported that > 365,000 nurses were employed across the country [5], making them the largest regulated body of health care professionals within Canada. In 2012 alone, the Canadian Federation of Nurses Unions reported that the cost of absenteeism due to illness, injury and disability in Canadian Nurses exceeded 734 million dollars, > 20 million dollars more than the cost of absenteeism in 2010 [6]. Nursing is associated with a wide variety of hazards, including various biological, chemical, and physical hazards that may cause occupational illness, as well as ergonomic and psychological hazards due to the highly demanding nature of the profession [7].

As occupational injury in the health care industry is a well-studied area, nurses are an ideal body of workers to examine when attempting to identify trends in lost-time injury incidents. In the Statistics Canada National Survey of the Work and Health of Nurses, it was identified that 45% of nurses surveyed were classified as overweight, and 14.4% were classified as obese on the body mass index (BMI) scale [8]. BMI uses a person's body weight in kg divided by the square of their height in m to obtain a number that can then be referenced to set standards. A BMI ≥ 25 kg/m² is considered to be overweight, and ≥ 30 kg/m² is considered to be obese by the World Health Organization [9].

In 2005, more than two million working Canadians were classified as obese [10], and with national obesity rates rising since then, it can be expected that the number is higher in the workforce today, with roughly 4.7 million Canadian adults' self-reported height and weight classifying them as obese on the BMI scale in 2012 [11]. Globally, it is expected that > 500 million adult men and women are also obese, with obesity rates doubling over the past 3 decades [9]. Obesity is defined by World Health Organization as “abnormal or excessive fat accumulation that may impair health” [9]. As those who are classified as obese are known to have higher levels of illness from diseases such as cancer and heart disease [9], as well as to suffer from some functional limitations due to their body weight, including reduced flexibility, limited range of movement, and decay in endurance [12], it is important to understand how obesity affects working Canadians, including nurses, especially when lost-time injuries are concerned.

There is a limited amount of current research directly examining BMI and obesity as predictors of lost-time injuries, although a fair amount of research has been done in the way of investigating lost-time and WC injuries, especially within the health care sector. The purpose of this paper is to examine the current literature surrounding lost-time injuries involving Registered Nurses (RNs), those where the RN has sought compensation through a WC board, and the predictors of these injuries, paying particular attention to those predictors that are linked to body weight and obesity. Understanding the predictors of lost-time illness and how they relate to obesity will help to provide more information on how obesity and high BMIs may affect workplace injury rates and workers' compensation claims and subsequent costs to the Canadian workforce and the economy. As health care, obesity, and occupational illness and injury are prevalent subjects beyond the Canadian border, findings from this study may help to increase interest and action surrounding obesity in the workplace abroad, specifically within the field of nursing, by demonstrating the need for prevention and intervention strategies focused on weight and weight management within the workplace, and may help to guide the creation and implementation of evidence-based prevention and intervention programs dealing with obesity and injury.

## 2 Materials and methods

Data were gathered using Google Scholar, Pub Med, Medline, CINAHL, PsycInfo, Social Science Abstracts, and Embase/Cochrane databases. Searches were conducted using the keywords and terms: lost-time injury, workers' compensation, occupational injury, workplace injury, injury, injuries, work, workplace, occupational, nurse, registered nurse, RN, health care, predictors, risk factors, risk, risks, cause, causes, obese, obesity, BMI, and body mass index. These searches were performed in May–August, 2014. Data were also gathered through utilizing the reference lists of articles retrieved using the aforementioned search terms to identify more material related to the topic. Current statistics on obesity and lost time injuries in Canada were gathered from online government resources. Nursing specific statistics were collected through the Canadian Federation of Nurses Unions and the Ontario College of Nursing.

For the purpose of this paper, only articles published within the past 10 years were utilized. Literature surrounding lost-time injuries involving RNs, those nurses in possession of a license from a national association such as the Canadian Nurses Association was examined. Studies where the RN has sought compensation through a workers' compensation board, and the predictors of these injuries were the main focus of this study, though other studies which measure injury or illness in other manners were also examined, though with caution. Research including measures and predictors linked to body weight, especially obesity were of particular interest and were included in this review.

**Fig. 1:**
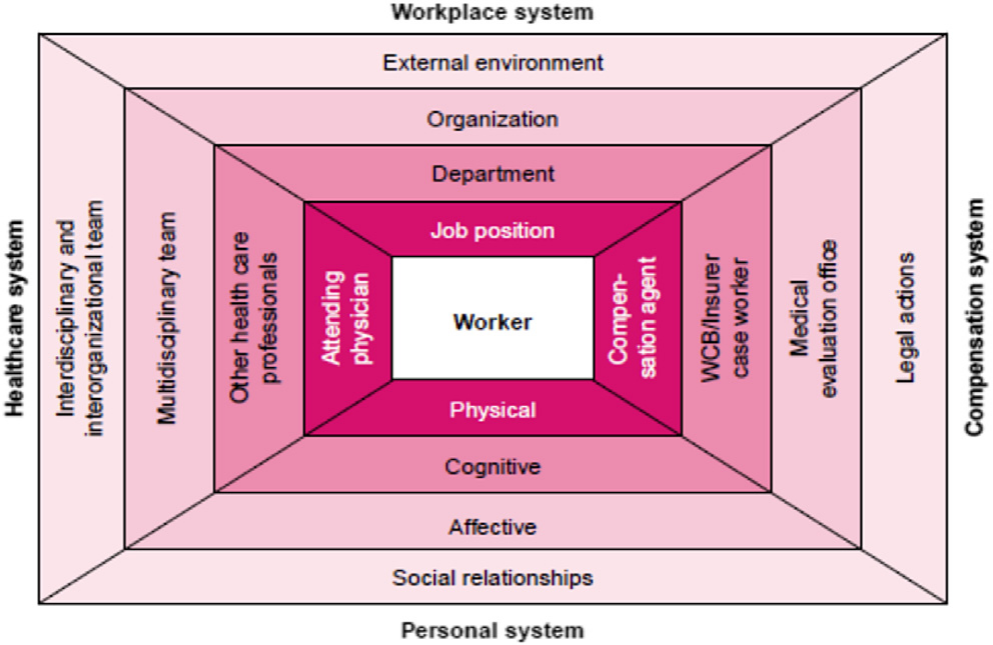
Work disability prevention management model [13].

Data on predictors or factors associated with lost-time injuries in nurses were gathered and organized using Loisel's Work Disability Prevention Management Model, illustrated in Fig. 1, to guide classification of the various factors identified.

### 2.1 Loisel's disability prevention management model

Workplace injury is a multifactorial problem, with injury and illness stemming from multiple conditions and causes of different varieties. This model was developed to facilitate a better understanding of the problem of occupational injury, specifically disability due to back pain, by stakeholders, as well as research on the subject [13]. This model acknowledges the contribution of factors related to the person, workplace, health care, and WC systems on the worker that may influence disability in the workplace. These factors must be acknowledged and dealt with in an organized manner if disability prevention is to be achieved [13].

To assist in better evaluating and understanding factors identified within the literature, Loisel's disability paradigm was used to classify predictors into two distinct categories, personal systems, and workplace systems, elements Loisel recognized must be considered when attempting to comprehend workplace injury. These systems interact with the health care system, as well as WC boards, and influence illness, injury, and disability at the worker level.

## 3 Review

A summary of literature reviewed on the topic of RNs and workplace injury is in Table 1. Other relevant studies addressed in this review involving other occupations are in Table 2.

In Whitaker's 2001 article examining the management of sickness absence, lost-time illness/injury/disease is operationalized as “an absence from work that is attributed to sickness by the employee and approved by the employer” [14]. In Canada, a time-loss injury is one for which a worker is compensated for their time off work following the injury, illness, or hazardous exposure, as defined by the Association of Worker Compensation Boards of Canada [15]. This could also include permanent leave from the workplace due to the injury or illness sustained while on the job. It was estimated in 2008 that less than one million occupational injury claims are reported each year to WC boards across the country [2], with about one-quarter of these being approved for compensation [3]. The health care industry represents the occupational group with the highest amount of WC claims across the country, with an increase in claim rates from 2011–2012 [3]. Therefore, analyzing the factors associated with these injuries and illnesses is important in furthering the understanding of possible occupational health and safety issues occurring in the industry.

## 4 Predictors of lost-time injury

Loisel's Disability Prevention Framework identifies the workplace system, as well as the personal system, which interact with the health care and compensation system in influencing injury, illness, and disability in the workplace [13].

### 4.1 Workplace system

The Workplace System involves factors and elements of the workplace environment, organization, department, and job position that may influence worker injury [13].

### 4.2 Job position

RNs are exposed to a vast array of occupational hazards, including various chemical, biological, physical, psychosocial, ergonomic, and safety hazards that may lead to injury or illness [7]. In a retrospective cohort study by Østbye et al [16] examining the relationship between BMI and number and types of WC claim-associated costs and lost workdays using data collected from 11,728 health care and university employees within the Duke University Health Surveillance System, it was found that those employed in occupations that require heavy lifting or have higher prevalence of ergonomic stress, such as nurses, had higher rates of workers compensation claims than those employed in lower risk, less strenuous occupations, such as science and administration, although this study did not focus on nurses specifically, but all employees within the health care network.

In a study of occupational and environmental risk factors for falls occurring in 2004–2007 among 2,300 health care workers using British Columbian WC board reporting databases, RNs were found to have experienced the most number of falls resulting in lost time injuries, more than any other health care job category identified. Those employed in positions that were described as requiring a great deal of physical activity and mobility were at an increased risk for sustaining a fall that results in a WC claim [17], though like the previous study, all occupations within the health care field were examined rather than focusing solely on registered nurses. Boyer et al's [18] study of 381 WC board claims from a single community hospital in Massachusetts in 2003–2005 also identified increased physical work as a risk factor for lost-time injury, noting that with every 10% increase on the physical workload scale, there was an approximate 23% greater chance of reporting a lost-time injury.

Qin et al [19] conducted a study aimed at determining the impact of workplace factors on the filing of WC claims in nursing home employees from 18 skilled nursing facilities in four US states. This included nurses who had filed a WC claim due to back pain in 2006–2009, and had completed at least one self-administered questionnaire around the time of the injury event. Job positions with high physical demands were associated with increased likelihood of filing a WC claim, and as physical demand increased, so did the likelihood of filing a claim, although once again, data retrieved and displayed were collected throughout various occupations within the health care facility and only included nurses in long-term care, only one subsector of nursing [19].

Finally, in a second study of the British Columbia workplace health and safety surveillance system and WC board data examining the demographics and workplace risk factors associated with serious falls, it was found that, although RNs sustained a lower proportion of falls that resulted in filing a WC claim due to a serious fall, the cost and duration of their claims were the highest of all health care worker positions [20], setting nurses apart from their peers.

Nursing is regarded as an occupation with many hazards, including mechanical and ergonomic hazards through activities such as lifting and moving patients, as well psychosocial hazards due to things like high work demands, shiftwork, and patient violence [7]. The data presented suggest that the physicality of a position in nursing not only increases the likelihood of injury above that of many other lower risk health care positions such as management and administration, but the severity and time lost due to that injury.

In Canada, the average RN has approximately 18 years of experience [5]. Risk of lost-time injury was found to increase with tenure for employees of the Duke University and Health System in a study of 5,082 nurses [21], although this overarching study included many types of nursing sectors, including gynecology, pediatrics, and emergency care. A similar trend was seen in a study of British Columbian health care workers, where incidence of lost-time injuries related to falls was seen to increase with years on the job [17]. A study of tertiary care workers by Pompeii et al [22] found nurses who worked < 5 years had three times greater risk of losing at least 8 work days due to illness or injury compared to those who had worked at the hospital for > > 10 years. Low tenure was also found to be a risk factor in a single hospital study by Boyer et al [18], who found that workers with < 2 years of tenure were 4.8 times greater chance of reporting an injury.

A broad range of nursing professions and environments means a multifarious workforce, with many different hazards in departmentally diverse positions. In Canada, the average age of a registered nurse is 45.2 years [5]. As age is closely linked to tenure in many situations, further implications of years at work will be discussed in the following sections.

### 4.3 Department

As aforementioned, there is a wide variety of nursing professions or specialties, many of which may be present in a single workplace. Many individual subsets of nursing have their own inherent set of risks, depending on the nature of the care, the patient being cared for, as well as the setting.

The study conducted by Rodríguez-Acosta et al [21] of the Duke University Health Care surveillance system records found that nurses who primarily worked with adults, and those employed in orthopedic and rehabilitation units within the system had higher risks of lost-time injury than those employed in other units, such as pediatrics or psychiatry. Studies of fall claims reported to the British Columbian WC board showed that those working in long-term care as having the highest rate and relative risk of falls [20], although those in acute care saw the highest number [17]. A study of reportable injuries logged by the occupational health departments at two academic Boston area hospitals showed that nurses employed in pediatric and neonatal units had the lowest injury rates of all units within the hospital [23]. Although this particular study did not examine lost-time injuries in particular, it supports the findings of the previously mentioned studies, showing that departments dealing with larger, adult patients are at a higher risk of injury than those dealing with children. In the 2005 National Survey of the Work and Health of Nurses (NSWHN), 25% of nurses reported experiencing back pain, compared with only 19% of the working population [8]. As heavy lifting is often associated with back pain, the type of patient and department are important factors when examining nursing injuries.

### 4.4 Organization

Organizational factors may include elements such as peer support, safety climate, and workplace training. In relation to peer support; 45% of female nurses and 51% of male nurses surveyed in the Canadian NSWHN felt that they had low coworker support, compared to only 33% of the general working population [8]. A study of ergonomic and socioeconomic risk factors for hospital workers' compensation injury claims found that low supervisory support was a risk factor for occupational injury. Increased psychological reward, as well as supervisor support decreased injury rates within the studied population [18]. In a study of 18 skilled long-term nursing facilities in four US states, social support was also associated with decreased claim rates [19]. Although limited data were found on the relationship between organizational environment, nurses, and lost-time injuries, studies of factors associated with non–lost-time injury in nurses' aides and direct care workers show that supportive work environments and positive organizational climate decreased risk of injury in workers surveyed [24].

### 4.5 Personal system

The personal system includes physical and psychosocial elements at the personal level, which may influence worker injury [13].

### 4.6 Psychosocial factors

Psychosocial factors can include many things, including cognitive elements, affective elements, as well as social relationships. In the 2005 NSWHN, 9% of nurses surveyed reported feeling depressed compared to 4–7% of the working population as a whole [8]. Information on psychosocial factors at the personal level in relation to nurse injury was limited, although associations can be made between psychosocial support at the organizational level and psychosocial wellbeing. At the personal level, psychosocial stress was found to be higher in nurses than their colleagues with similar salary and education levels [25]. High psychosocial demand was found to be associated with increased claim likelihood in health care workers [18], and feeling nervous was associated with lost time injuries in a single study of tertiary care workers [22]. As nursing presents itself with various psychosocial hazards, such as intense periods of stress, workplace violence, and exposure to traumatic events [7], mental health is an important factor when looking in to the overall health and wellbeing of nurses and should not be forgotten as an a potential influence on workplace injury and disease.

### 4.7 Physical factors

When exploring the interaction between nurses and lost-time injury and illness, physical factors are important considerations, especially due to the high physicality of the job.

Although not necessarily a physical feature, age is an important physical factor in the workforce. In 2012, 25% of nurses employed within Canada were aged ≥ 50 years [5]. Many studies of lost-time injuries and illness within the health care sector define age as a risk factor for occupational injury. In the literature examined for the purpose of this study, most researchers found that workers aged ≥ 40–60 years have increased rates of occupational injury resulting in lost time. Health care workers aged ≥ 50–60 years were found to have the highest risk of injury resulting in a time loss injury in studies conducted on Duke University Health Surveillance System data [16], as well as studies of BC workers’ compensation claim data [19], and US Midwest nursing care facility workers [26]. Drebit et al [17] found that 68% of falls resulting in lost-time injuries in BC health care workers between 2004 and 2007 were in employees aged 40–60 years. This study also found that fall claims rose as age increased along with tenure.

Current research regarding health related risk factors, such as smoking, high blood pressure, weight, and physical activity is limited not only within the health care sector, but across the board. In Østbye et al's [16] study of the Duke Health and Safety Surveillance System, a clear relationship was found between BMI category and claims rates in health care employees, with claims rates for the heaviest employees being twice that of those employees of a normal weight. Cost and number of days lost also increased rapidly as BMI increased in health care employees [16]. Finally, those employees surveyed with a BMI of ≥ 30 and employed in a high-risk occupation with high physical demands and ergonomic stress had a claims risk ratio of 7.04 compared to employees of normal weight employed in lower risk positions. Injuries most often associated with the higher BMI categories included injury to the back, wrist, neck, shoulders, and lower extremities [16].

Studies of BMI and its relationship to occupational injury in other industries have discovered similar findings, with high BMI being related to increased lost-time injuries in firefighters [27], although more research is needed in the area of employee health and potential risk factors such as high blood pressure and physical activity and their effect on lost-time injuries before the relationship can be fully understood.

Despite limited evidence on the definite effects of BMI and obesity on lost-time injuries in nurses and the health care sector, by using information drawn from the literature as well as Canadian workplace injury and obesity statistics, interesting parallels can be seen between factors found within the literature and current information on obesity. As noted earlier, 45% of Canadian nurses surveyed were considered to be overweight in 2005, and 14.4% were considered to be obese [8]. In 2005, the percentage of female nurses who were classified as overweight was significantly higher than the general employed female population [8]. With obesity rates on the rise, it could be expected that current values would be higher.

In Canada, obesity is most prevalent in older workers aged 55–64 years [10], ages that are associated with higher rates of lost-time injury in nurses and other health care workers [16,17,20,21,26].

Higher proportions of obese workers report having high job strain, including high psychological demands and feelings of low social support [10], factors also found to be associated with increased lost-time injuries in health care. As noted by Park, it is hard to determine temporal ordering in this situation, as one factor may precede another [10].

Obesity is associated with various functional limitations [12] and increased risk of poor health [9]. Although it was shown that risk factors for injury seem to differ between departments, physical demands, and ergonomic strain can be seen as a common theme [16–23,25]. Those suffering from poor health and limited mobility, flexibility, low endurance, or other conditions often associated with obesity would be thought to be more likely to sustain an injury while performing highly physical tasks, such as lifting and assisting adult patients, due to their physical limitations, even though the literature presented does not make these direct links. Obesity may also affect nurses this way via a different route, through having a higher number of heavier patients in their care, resulting in increased strain on the job.

Current data specifically focused on obesity as a predictor for lost-time injury are very limited. Generalized data on obesity in the workplace were used to speculate upon the relationship between lost-time injury factors identified in the literature and BMI. Only data regarding RNs, those with RN designations, were included in this study. The studies examined also described lost-time injuries in nurses' aides, or the health care sector as a whole, and often made generalizations regarding the findings. Although some studies reporting data taken from health care settings present results in a manner that allow only data taken from RNs to be extracted, many report results as broad findings of a generalized health care sector, making it challenging to determine whether the values given were representative of nursing staff. Several studies relied on the same data sets to come up with their findings. Only English articles were retrieved and, although definitions and paradigms were drawn from older literature, studies on workplace injury obtained were limited in publication to the past 10 years.

## 5 Conclusion

There is a definite lack of evidence surrounding the influence of health factors such as obesity on lost-time injuries both for nurses within the health care field, as well as in the general working population. Nurses present a diverse subsection of the health care sector, employed in an occupation with many different risks. As the health care sector continues to remain the leading claimant of lost-time injuries in Canada with claim rates increasing from 2011 to 2012 [3], ensuring attention is paid to occupational issues within the health care sector is important. Obesity and occupational injury are worldwide concerns, afflicting hundreds of millions of people around each year [1,9]. Better examining factors that may lead to increased rates of illness and injury, including both workplace factors, as well as personal factors such as physical and mental health, will be important in helping to lessen the burden of occupational disease in the health care sector, as well as other occupational settings. Continued study on these personal or health related factors can help to support the creation and implementation of evidence-based prevention and intervention programs aimed at health promotion and weight management in the workplace and potentially beyond, a necessity should the rate of obesity continue to rise.

## Conflicts of interest

The authors declare that they have no competing interests.

## Figures and Tables

**Table 1:** Workplace injury in registered nurses (RNs)

Refs	Purpose	Study design	Participants	Interventions	Findings	Limitations
**Alamgir et al 2011****[20]**	To examine the demographic and workplace risk factors of serious falls and associated economic burden in Canadian health care workers	Fall injury data obtained from 2005–2008 from BC workplace health and safety surveillance system and linked with WC claims and payroll records	*n* = 938 fall injury claims from BC health care workers (including workers in hospitals, community care, long-term care). One third of study population were RNs	No interventions	Workers aged > 60 y, those employed part-time, or employed in long-term care sector sustained more serious falls. Females, long-term care workers, RNs, care aids, and maintenance workers had most costly falls	Study only accounted for incidents that received compensation and only captured falls among one health region's workers
**Bell et al 2013****[26]**	To describe the slip, trip, and fall injury experience and trends in a population of nursing home workers, identify risk factors for slip trip and fall injuries, and develop prevention strategies	WC injury claims data, narrative information on the injury and payroll data from 1996–2003 obtained and analyzed from 6 US Midwest nursing homes	*n* = 86 slip, trip, and fall related WC claims	No interventions	Nursing home workers experience more slip, trip, and fall-related injury claims than workers in other industries. Most slips, trips and falls were attributed to hazards that could be controlled. Workers aged > 50 y experience more falls	Study included a greater proportion of care aides than nurses. Results were generalized across job types. Small sample size, only injuries compensated were included
**Boden et al 2012****[23]**	To study risk of injury in patient care workers using OSHA injury definitions, comparing nurses and nurses' aides	Records from human resources and occupational health service databases	*n* = 5,991 nurses and *n* = 1,543 aides at two large academic hospitals in the Boston area	No interventions	Back injuries were more common than any other injuries. Over exertion was the major cause of days away from work. Nurses with < 5 y job tenure had lower days away rates. Men had lower injury rates. Nurses in the operating room and in the float pool (moved between units) had more days-away injuries. Lowest rates were in neonatal and pediatric units and units with little patient handling	Injuries identified by OSHA records as “days away” and “no days away” rather than compensated lost-time injuries. Hospitals studied provided transitional work, wage continuation that may affect return outcomes. Administration data did not allow for understanding of causes, under reporting, individual workplace factors
**Boyer et. al 2009****[18]**	To describe frequency of hospital WC claims by SES, estimate the likelihood of WC claims associated with physical workload, work organization and psychosocial exposure, and explore the degree ergonomic exposures explain difference in relative risks associated with SES	Hospital administrative and WC data 2003–2005 obtained and analyzed. Employees were classified by SES by education, responsibility required for position. A job exposure matrix was developed and used to provide information about working conditions	*n* = 1,483 total employees of a Massachusetts community hospital. *n* = 358 nurses.	No interventions	Jobs with highest injury rates included nurses, semi-professionals, and semi-skilled. Increased physical work, psychological demands and low job tenure, low psychosocial reward, low supervisor support increased risk	Study was not able to match all WC cases to SES categories, did not access individual info beyond age, race, gender, tenure, measure of social factors only included one question, injury risk estimates may be biased due to underreporting
**D'Errico et al 2007****[25]**	To describe the risk of work injury by SES in hospital workers. To assess whether SES gradient in injury risk is explained by differences in psychosocial, ergonomic or organizational factors	Administrative data and OSHA injury logs from 1997–2002 were obtained and analyzed from two hospitals in Massachusetts. Jobs were classified into 5 SES categories by job title. Psychosocial, ergonomic and organizational exposures were identified using a job exposure matrix	*n* = 3074 hospital workers	No interventions	Injury risk highest among semi-skilled workers and nurses. Predictors of risk of injury included decision latitude, supervisor support, force exertion and temperature extremes	May have been underreporting of injury, job exposure matrix used may not account for variability in exposures, did not evaluate injury at the individual level, therefore did not look at potential confounding factors, extraoccupational risk factors, SES not based on household income or other SES indicators. Possible errors in job denominators
**Drebit et al 2010****[17]**	To examine risk factors, including occupation type, workplace design, work setting, organization and environmental conditions in a large health care worker population	Data on falls occurring in 2004–2007 was collected and analyzed from WC claims, internal injury database, and payroll records from BC's health care sector	*n* = 23,000 employees total. *n* = 411 falls identified	No interventions	Majority of falls occurred in acute care, females, workers aged 40–60 y. RNs experienced the most number of falls. Fall rates highest in long-term care. Increase in fall rate with age and experience. Results varied greatly by sector, occupation	Analysis was short-term, unable to look at individual physical and behavioral risks, management factors, organization safety culture, and other extrinsic factors. Data based on employee description of incident, only time loss injuries included, possible under reporting of injuries
**Østbye et al 2007****[16]**	To determine the relationship between BMI and number and type of workers' compensation claims, costs, and lost workdays	Retrospective cohort study. Data collected from WC claims and Duke Health and Safety surveillance system	*n* = 11,728 health care and university employees from Duke University and Duke University Health System. *n* = 1,161 nurses	No interventions	Clear linear relationship between BMI and claim rates. Combination of high-risk occupation and obesity particularly detrimental. Higher relative risk for employees aged > 55 y, low tenure	BMI was only available for employees who had completed a health risk assessment, did not include information on shift work. Potential unobservable factors, false claims
**Pompeii et al 2010****[22]**	To examine possible predictors of lost workdays among nurses and nurses' aides who sought treatment for work-related back pain	Data collected from clinic surveys administered for nursing personnel during initial treatment after injury in 1994–2006, as well as employee health records	*n* = 589 total nursing employees from Duke University Medical Center who sought treatment through the company health clinic and completed the back-injury survey *n* = 430 nurses	No interventions	Working < 5 y in the hospital was predictive of losing > 8 workdays. Majority of injuries occurred in nurses working in medical and surgical inpatient units and intensive care	Injuries were not WC injuries; recorded as < 7 or > 8 workdays missed. No information on injured who did not fill out a survey or had no medical records available. Demographic information was not collected
**Qin et al 2014****[19]**	To determine the impact of workplace factors on filing of WC claims among nursing home workers	Data collected from self-administered questionnaires distributed in 2006–2009 in 18 skilled nursing facilities in the US and matched to WC claims	*n* = 2,639 employees who returned at least one survey *n* = 238 nurses	No-lift policy (effects not measured by study)	Higher physical demands at work increased likelihood of filing a claim. Higher levels of social support, education and BMI decreased the likelihood of filing for nurses	Only small number of employees who reported back-pain in questionnaire filed for WC. Possible underreporting, recall bias. Difficult to tell if back-pain caused by work from survey. High staff turnover rate over study period. All nursing homes owned and managed by single company
**Rodriguez-Acosta et al 2009****[21]**	To assess risk of work-related injuries in an acute care setting while contrasting injuries of aides and nurses	Retrospective cohort studies of US nurses and aides working in acute care 1997–2004. Data collected from personnel files and WC records	*n* = 5,083 nurses and *n* = 1,689 aides from Duke University Hospital and Durham Regional Hospital	No interventions	Most injuries for both groups were a result of delivering direct patient care. Greater risk for female nurses, aged > 60 y, those working in orthopedics and rehabilitation units, units with adult patients. Lifting was leading cause of injury	Probably some error involved in claim data due to underreporting. Health surveillance system does not contain exposure data for physical and psychological demands, individual risk factors, members not experiencing injury

BC, British Columbia; BMI, body mass index; OSHA, Occupational Safety and Health Administration; SES, socioeconomic status; WC, worker compensation.

**Table 2:** Workplace injury in other occupations

Reference	Purpose	Study design	Participants	Interventions	Findings	Limitations
**Kuehl et al 2012****[27]**	To determine the relationship between lifestyle variables including BMI and filing a WC claim due to fire fighter injury	Cross-sectional evaluation of firefighter injury related to WC claims and medical history, physical survey occurring 5 years after a health intervention study	*n* = 433 firefighters from Oregon and Washington	Health intervention was conducted 5 years prior to study	The odds of filing a WC claim were 3 times higher for obese firefighters than normal BMI	Only observed firefighters. BMI may not be good measure for those with high muscle mass
**McCaughey et al 2014****[24]**	To explore the relationship between nursing assistant injury rates and key outcomes while exploring work environment factors that can decrease the rates of workplace injury	Data was collected from 2004 National Nursing Assistant Survey and analyzed	*n* = 3,017 assistants from 592 nursing homes across the USA	No interventions	Assistants who experienced workplace injuries had low levels of job satisfaction, increased turnover intentions. Injuries were related to low ratings in prevention training, supervisor support, employee engagement, and training	Only information from nurses assistants, older data set may not be current. Survey was not specifically designed to evaluate worker injury and training. Data from single time period, cannot establish temporal ordering. Injuries were self-reported, not specified as lost-time injuries

BMI, body mass index; WC, worker compensation.
